# Supplementary material for: Factors Impacting Range Hood Use in California Houses and Low-Income Apartments
Source: Int J Environ Res Public Health. 2020 Nov 28;17(23):8870. doi: 10.3390/ijerph17238870 (PMC7729668; doi:10.3390/ijerph17238870)
Supplement: Supplementary file 1 [file ijerph-17-08870-s001.pdf]

Supporting Information for:

Factors Impacting Range Hood Use in California Houses and Low-Income Apartments

Haoran Zhao[1], Wanyu R. Chan[1], William W. Delp[1], Hao Tang[2], Iain S. Walker[1], Brett C. Singer[1*]

[1] Residential Building Systems Group and Indoor Environment Group, Lawrence Berkeley National Laboratory, Berkeley, CA, 94720, USA; haoranzhao@lbl.gov

[2] National Centre for International Research of Low-carbon and Green Buildings, Ministry of Science and Technology, Chongqing University, Chongqing 400045, China

* Correspondence: bcsinger@lbl.gov; wrchan@lbl.gov

Tables

Table S1. Time delays in range hood start.. 2
Table S2. Range hood use duration as a percentage of cooking time.. 2
Table S3. Investigation of associations between equipment and home characteristics and rate of range hood use by home, for houses and apartments... 3
Table S4. Investigation of associations between household characteristics and rate of range hood use across all the cooking events for all homes. 4
Table S5. Investigation of associations between household characteristics and rate of range hood use across all the cooking events for houses only.. 5
Table S6. Investigation of associations between household characteristics and rate of range hood use across all the cooking events for apartment only. 6
Table S7. Range hood use by cooking type: Any cooktop use vs. only oven use.......................... 7
Table S8. Range hood use by cooking type: Only cooktop use vs. any oven use 8
Table S9. Range hood use by cooking type: Only cooktop use vs. only oven use......................... 8
Table S10. Range hood use by cooking type: Any cooktop use vs. any oven use 9
Table S11. Range hood use by cooking type for houses with ovens located underneath the range hood.. 10
Table S12. Range hood use by oven use duration 10

In this study, we defined "full use" as the range hood starting within 3 minutes of cooking and being used for >80% of the cooking time. Table S1 shows the counts and percentages of cooking events with range hood use categorized by their delays in start time. Table S2 shows the counts and percentages of cooking events where range hood usage was calculated as % of cooking time.

Table S1. Time delays in range hood start

Range hood start delay	Cooking event (%)
≤3 minutes	137 (52%)
3–10 minutes	58 (22%)
>10 minutes	68 (26%)

Table S2. Range hood use duration as a percentage of cooking time

Range hood use as % of cooking time	Cooking event (%)
≥80%	107 (41%)
50–80%	53 (20%)
<50%	103 (39%)

Table S3. Investigation of associations between equipment and home characteristics and rate of range hood use by home, for houses and apartments.

	P-value[1]		
Home character	All home (N=53)	House (N=37)	Apartment (N=16)
Floor area	0.91	0.99	0.24
Number of occupants	0.73	0.12	**0.09**[2]
Density	0.46	0.19	0.14
ACH	0.37	0.95	0.18
ACH/person	0.96	0.33	0.55
ACH/floor area	0.96	0.86	0.30
Installed hood airflow low speed	0.27	0.43	0.12
Installed hood airflow high speed	0.29	0.22	0.69
Certificate sound level low speed	0.33	0.13	0.42
Certificate sound level high speed	0.29	0.22	0.69

[1]P-value was calculated using ANOVA between hood use rate in each home and home characters
[2]Lower range hood use rate in apartments with higher occupancy

Table S4. Investigation of associations between household characteristics and rate of range hood use across all the cooking events for all homes.

All home		Homes with answer "yes"			Homes with answer "no"		
**Household character category**	**P-value**	**Cooking events**	**Any hood use**	**% Use**	**Cooking events**	**Any hood use**	**% Use**
Has a child	0.51	335	109	0.33	425	148	0.35
Has a senior	0.19	166	49	0.30	594	208	0.35
Has a child/senior	0.06	400	123	0.31	360	134	0.37
Has health concerns	0.08	479	150	0.31	274	103	0.38
Insufficient air movement indoors	**0.01**	**224**	**60**	**0.27**	**499**	**185**	**0.37**
Too much air movement indoors	0.17	149	58	0.39	565	186	0.33
Vacuum frequency ≥ few times/week	**<0.01**	**263**	**58**	**0.22**	**486**	**192**	**0.40**
Windows open >2h/day in 3 seasons	0.27	237	73	0.31	516	180	0.35
Reason for not using a hood: forget	**<0.01**	**225**	**87**	**0.39**	**381**	**96**	**0.25**
Reason for not using a hood: no need	0.10	252	67	0.27	354	116	0.33
Reason for not using a hood: noisy	0.32	186	51	0.27	420	132	0.31
Reason for not using a hood: ineffective	**<0.01**	**69**	**7**	**0.10**	**537**	**176**	**0.33**
Reason for not using a hood: open window	0.10	165	58	0.35	441	125	0.28
Has some college or higher degree	**0.00**	**682**	**240**	**0.35**	**66**	**10**	**0.15**
Has bachelor or higher degree	**0.01**	**542**	**196**	**0.36**	**206**	**54**	**0.26**
Has graduate degree	**<0.01**	**394**	**151**	**0.38**	**354**	**99**	**0.28**
Household income≥35k	**<0.01**	**593**	**211**	**0.36**	**136**	**29**	**0.21**
Household income≥75k	**0.01**	**524**	**188**	**0.36**	**205**	**52**	**0.25**
Household income≥100k	**0.02**	**482**	**173**	**0.36**	**247**	**67**	**0.27**
Household income≥150k	0.11	212	79	0.37	517	161	0.31

P-value was calculated using Chi-square test for hood use between two household character categories

Table S5. Investigation of associations between household characteristics and rate of range hood use across all the cooking events for houses only.

Houses		Houses with answer "yes"			Houses with answer "no"		
**Household character category**	**P-value**	**Cooking events**	**Any hood use**	**% Use**	**Cooking events**	**Any hood use**	**% Use**
Has a child	0.97	273	98	0.36	297	107	0.36
Has a senior	0.53	145	49	0.34	425	156	0.37
Has a child/senior	0.09	338	112	0.33	232	93	0.40
Has health concerns	0.60	356	130	0.37	207	71	0.34
Insufficient air movement indoors	0.22	151	48	0.32	388	145	0.37
Too much air movement indoors	0.45	135	52	0.39	404	141	0.35
Vacuum frequency ≥ few times/week	0.02	140	38	0.27	419	160	0.38
Windows open >2h/day in 3 seasons	0.19	155	62	0.40	408	139	0.34
Reason for not using a hood: forget	0.01	206	80	0.39	244	68	0.28
Reason for not using a hood: no need	**<0.01**	**241**	**64**	**0.27**	**209**	**84**	**0.40**
Reason for not using a hood: noisy	0.20	171	50	0.29	279	98	0.35
Reason for not using a hood: ineffective	**0.02**	**42**	**7**	**0.17**	**408**	**141**	**0.35**
Reason for not using a hood: open window	0.11	110	43	0.39	340	105	0.31
Has some college or higher degree	NA						
Has bachelor or higher degree	0.50	515	186	0.36	48	15	0.31
Has graduate degree	**0.05**	**394**	**151**	**0.38**	**169**	**50**	**0.30**
Household income≥35k	NA						
Household income≥75k	0.93	524	188	0.36	37	13	0.35
Household income≥100k	0.94	482	173	0.36	79	28	0.35
Household income≥150k	0.58	212	79	0.37	349	122	0.35

P-value was calculated using Chi-square test for hood use between two household character categories

Table S6. Investigation of associations between household characteristics and rate of range hood use across all the cooking events for apartment only.

Apartments		Houses with answer "yes"			Houses with answer "no"		
**Household character category**	**P-value**	**Cooking events**	**Any hood use**	**% Use**	**Cooking events**	**Any hood use**	**% Use**
Has a child	0.04	62	11	0.18	128	41	0.32
Has a senior	<0.01	21	0	0.00	169	52	0.31
Has a child/senior	0.04	62	11	0.18	128	41	0.32
Has health concerns	<0.01	123	20	0.16	67	32	0.48
Insufficient air movement indoors	**<0.01**	**73**	**12**	**0.16**	**111**	**40**	**0.36**
Too much air movement indoors	0.24	14	6	0.43	161	45	0.28
Vacuum frequency ≥ few times/week	**<0.01**	**123**	**20**	**0.16**	**67**	**32**	**0.48**
Windows open >2h/day in 3 seasons	**<0.01**	**82**	**11**	**0.13**	**108**	**41**	**0.38**
Reason for not using a hood: forget	0.08	19	7	0.37	102	19	0.19
Reason for not using a hood: no need	0.62	11	3	0.27	110	23	0.21
Reason for not using a hood: noisy	0.14	15	1	0.07	106	25	0.24
Reason for not using a hood: ineffective	**<0.01**	**27**	**0**	**0.00**	**94**	**26**	**0.28**
Reason for not using a hood: open window	0.16	55	15	0.27	66	11	0.17
Has some college or higher degree	**0.01**	**119**	**39**	**0.33**	**66**	**10**	**0.15**
Has bachelor or higher degree	0.18	27	10	0.37	158	39	0.25
Has graduate degree	NA						
Household income≥35k	0.23	32	10	0.31	136	29	0.21
Household income≥75k	NA						

P-value was calculated using Chi-square test for hood use between two household character categories

Table 1 in the main text found that in single family houses, residents are more likely to use range hood or OTR when cooking with cooktop only, compared to oven only. But this difference is not statistically significant for the apartments. Because ovens and cooktop burners can be used together in a cooking event, we compared full range hood use and any range hood use for different combinations of burners: any cooktop use vs. only oven use, only cooktop use vs. any oven use, only cooktop use vs. only oven use and any cooktop use vs. any oven use. The results are shown in Table S7-S10 for houses and apartments, analyzed separately and together. The overall results show that a range hood or OTR was likely used more frequently when residents used a cooktop burner either alone, or together with an oven burner, compared to use of the oven alone, or with a cooktop in single family houses. Residents in apartments operated range hoods slightly more frequently when using the oven with or without a cooktop burner, but the differences were not statistically significant.

Table S7. Range hood use by cooking type: Any cooktop use vs. only oven use

	**Cooking type**	**Cooking events**	**Any hood use**	**% Use**	**Full hood use**	**% Use**
House	Any CT (CT+CTOV)	526	193	37%	63	12%
	OV only	48	12	25%	7	15%
	p-value		0.11		0.37	
Apartment	Any CT (CT+CTOV)	195	53	27%	15	8%
	OV only	15	5	33%	0	0%
	p-value		0.60		0.27	
House+ apartment	Any CT (CT+CTOV)	721	246	34%	78	11%
	OV only	63	17	27%	7	11%
	p-value		0.25		0.94	

P-value was calculated using Chi-square test for hood use between two cooking types

Table S8. Range hood use by cooking type: Only cooktop use vs. any oven use

	Cooking type	Cooking events	Any hood use	% Use	Full hood use	% Use
House	CT only	487	182	37%	61	13%
	Any oven (CTOV+OV)	87	23	26%	9	10%
	p-value		**0.05**		0.57	
Apartment	CT only	190	50	26%	14	7%
	Any oven (CTOV+OV)	20	8	40%	1	5%
	p-value		0.19		0.70	
House+ apartment	CT only	677	232	34%	75	11%
	Any oven (CTOV+OV)	107	31	29%	10	9%
	p-value		0.28		0.59	

P-value was calculated using Chi-square test for hood use between two cooking types

Table S9. Range hood use by cooking type: Only cooktop use vs. only oven use

	Cooking type	Cooking events	Any hood use	% Use	Full hood use	% Use
House	CT only	487	182	37%	61	13%
	OV only	48	12	25%	7	15%
	p-value		**0.09**		0.68	
Apartment	CT only	190	50	26%	14	7%
	OV only	15	5	33%	0	0%
	p-value		0.56		0.28	
House+ apartment	CT only	677	232	34%	75	11%
	OV only	63	17	27%	7	11%
	p-value		0.24		0.99	

P-value was calculated using Chi-square test for hood use between two cooking types

Table S10. Range hood use by cooking type: Any cooktop use vs. any oven use

	Cooking type	Cooking events	Any hood use	% Use	Full hood use	% Use
House	Any CT (CT+CTOV))	526	193	37%	63	12%
	Any oven (OV+CTOV)	87	23	26%	9	10%
	p-value		**0.07**		0.86	
Apartment	Any CT (CT+CTOV))	195	53	27%	15	8%
	Any oven (OV+CTOV)	20	8	40%	1	5%
	p-value		0.30		0.99	
House+ apartment	Any CT (CT+CTOV))	721	246	34%	78	11%
	Any oven (OV+CTOV)	107	31	29%	10	9%
	p-value		0.32		0.74	

P-value was calculated using Chi-square test for hood use between two cooking types

In single family houses, some ovens were located underneath the range hood (N=32), while others had separate ovens located off to a side (N=22). Table S11 repeats the above analysis (Table S7–S10) for the single family homes that had ovens located underneath the range hood. For that subset of houses, there was no statistically significant difference in range hood use between cooking with cooktop burners and ovens. This suggests the finding that residents in houses used range hood more frequently when cooking with cooktop compared to oven may be explained by some houses having separate ovens located off to a side, where the hood use was less frequent (see Table 4).

Table S11. Range hood use by cooking type for houses with ovens located underneath the range hood

	Cooking type	Cooking events	Any hood use	% Use	Full hood use	% Use
House	Any CT (CT+CTOV)	314	116	37%	41	13%
	OV only	48	12	25%	7	15%
	p-value		0.14		0.82	
House	CT only	298	109	37%	41	14%
	Any oven (CTOV+OV)	34	14	41%	5	15%
	p-value		0.60		0.88	
House	CT only	298	109	37%	41	14%
	OV only	18	7	39%	5	28%
	p-value		0.81		0.16	
House	Any CT (CT+CTOV)	314	116	37%	41	13%
	Any OV (OV+CTOV)	34	14	41%	5	15%
	p-value		0.71		0.79	

P-value was calculated using Chi-square test for hood use between two cooking types

Table S12 shows the relationship between range hood use and oven use duration. The analysis was performed separately for ovens located underneath the range hood, and ovens located off to a side. No statistically significant relationship was found.

Table S12. Range hood use by oven use duration

Oven use (minutes)	Oven underneath range hood			Oven off to a side		
	Cooking events – OV only	Any hood use	% Use	Cooking events – OV only	Any hood use	% Use
1-40	15	7	47%	12	1	8%
>40	18	5	28%	18	4	22%
p-value	0.26			0.32		

P-value was calculated using Chi-square test for hood use comparing different oven use duration
